# Small-molecule inhibitors of kinases in breast cancer therapy: recent advances, opportunities, and challenges

**DOI:** 10.3389/fphar.2023.1244597

**Published:** 2023-08-30

**Authors:** Isha Bansal, Amit Kumar Pandey, Munindra Ruwali

**Affiliations:** ^1^ Amity Institute of Biotechnology, Amity University Haryana, Gurugram, Haryana, India; ^2^ Department of Biotechnology, National Institute of Pharmaceutical Education and Research (NIPER-Ahmedabad), Gandhinagar, Gujarat, India

**Keywords:** breast cancer, small-molecule inhibitors, kinases, drug resistance, therapy

## Abstract

Breast cancer is the most common malignancy in women worldwide and despite significant advancements in detection, treatment, and management of cancer, it is still the leading cause of malignancy related deaths in women. Understanding the fundamental biology of breast cancer and creating fresh diagnostic and therapeutic strategies have gained renewed focus in recent studies. In the onset and spread of breast cancer, a group of enzymes known as kinases are extremely important. Small-molecule kinase inhibitors have become a promising class of medications for the treatment of breast cancer owing to their capacity to specifically target kinases involved in the growth and progression of cancer. The creation of targeted treatments that block these kinases and the signalling pathways that they activate has completely changed how breast cancer is treated. Many of these targeted treatments have been approved for the treatment of breast cancer as clinical trials have demonstrated their great efficacy. CDK4/6 inhibitors, like palbociclib, abemaciclib, and ribociclib, EGFR inhibitors such as gefitinib and erlotinib and HER2-targeting small-molecule kinases like neratinib and tucatinib are some examples that have shown potential in treating breast cancer. Yet, there are still difficulties in the development of targeted medicines for breast cancer, such as figuring out which patient subgroups may benefit from these therapies and dealing with drug resistance problems. Notwithstanding these difficulties, kinase-targeted treatments for breast cancer still have a lot of potential. The development of tailored medicines will continue to be fuelled by the identification of novel targets and biomarkers for breast cancer as a result of advancements in genomic and proteomic technology.

## 1 Introduction

Breast cancer is one of the most prevalent malignancies in women worldwide accounting for 25% of all female cancers (Sung et al., 2021). Breast lobules, fatty tissue, milk ducts, and other breast structures, all can develop into breast cancer. In the circulation or lymphatic system, the disease can spread to different body sites. Breast cancer has a complex etiology that includes both hereditary and environmental components (Łukasiewicz et al., 2021). Personal history of breast cancer, dense breast tissue, age, family history, specific genetic abnormalities like BRCA1 and BRCA2, and oestrogen exposure are important risk factors (Feng et al., 2018). Early detection by screening, like mammography, can enhance treatment results and raise the likelihood of survival. Depending on the stage and characteristics of the tumour, breast cancer is treated with a mix of surgery, radiation therapy, chemotherapy, hormone therapy, and targeted therapy (Cardoso et al., 2020). More individualized therapy approaches that consider the unique qualities of the patient and the tumor have acquired popularity in recent years (Rugo et al., 2016).

Understanding the fundamental biology of breast cancer and creating fresh diagnostic and therapeutic strategies have gained renewed focus in recent studies. Some unique genetic abnormalities, such as in PALB2 and ATM, that raise the risk of breast cancer have also been recently identified (Goldgar et al., 2011; Kwong et al., 2021). Immunotherapy is being researched as a potential treatment for breast cancer since it uses the body’s immune system to fight cancer cells (Mittendorf et al., 2014). The most recent developments in immunotherapy for breast cancer include vaccine-based methods, checkpoint inhibitors, and chimeric antigen receptor T cells (Debien et al., 2023). The developments in tailored therapy options, such as targeted treatments and immunotherapies, for various subtypes of breast cancer have also been highlighted recently (Masoud and Pagès, 2017).

In the onset and spread of breast cancer, a group of enzymes known as kinases are extremely important. They catalyse the transfer of phosphate groups from ATP to specific amino acids on target proteins. For instance, breast cancer usually activates protein kinase B (Akt), which increases cell invasion, survival, and proliferation. As a result, it is desirable to target protein kinases like Akt in the therapy of breast cancer (Carvajal and Manfredi, 2013). Uncontrolled cell proliferation and survival, a defining feature of cancer, can result from the dysregulation of kinase signalling pathways. For instance, the HER2 kinase is activated in aggressive breast cancer subtypes and is linked to a poor prognosis (Moasser, 2007). Likewise, breast cancer frequently has mutations in the PI3K and AKT kinases, which promote tumour growth and therapeutic resistance (Engelman et al., 2006). Attacking kinases with specific inhibitors has demonstrated substantial clinical advantage in the treatment of breast cancer, especially when used in conjunction with other medicines (Masoud and Pagès, 2017). Furthermore, recent studies indicate that CDK4/6 is a crucial player in the initiation and development of breast cancer (Niu et al., 2019). Several clinical trials have demonstrated a considerable therapeutic advantage for the use of CDK4/6 inhibitors, like palbociclib, in the treatment of advanced breast cancer that is hormone receptor-positive but HER2-negative (Finn et al., 2016). Moreover, additional kinases like JAK and SRC are also implicated in the emergence and development of breast cancer. Basal-like breast cancer frequently exhibits dysregulation of the signalling system, which promotes cell growth and inflammation. Additionally, increase tumour invasiveness and metastasis have been linked to SRC kinase overexpression (Luo et al., 2022). In preclinical research and clinical trials, targeting these kinases with certain inhibitors yielded positive outcomes (Jain et al., 2015; Pernas and Tolaney, 2019).

Small-molecule kinase inhibitors have become a promising class of medications for the treatment of breast cancer owing to their capacity to specifically target kinases involved in the growth and progression of cancer. The HER2 kinase inhibitors lapatinib and tucatinib, as well as the CDK4/6 inhibitors ribociclib and palbociclib, have all been given the green light for the treatment of breast cancer. These medications have significantly increased patients’ overall survival with advanced breast cancer in multiple clinical trials, especially when used in conjunction with other treatments (Finn et al., 2016; Tripathy et al., 2018). In addition, further studies seek to discover fresh targets for kinase inhibitors and devise strategies for overcoming therapeutic resistance. In patients with hormone receptor-positive and HER2-negative advanced breast cancer, these medications have shown to have a considerable therapeutic effect, including enhanced overall survival and progression-free survival (Sledge et al., 2020). HER2-targeting small-molecule kinases like neratinib and tucatinib have demonstrated promising outcomes in the treatment of HER2-positive breast cancer, especially in patients with brain metastases (Murthy et al., 2020; Curigliano et al., 2022). Moreover, research is being done to find new targets for small-molecule kinase inhibitors, such as the commonly dysregulated PI3K/AKT/mTOR pathway or the FGFR pathway (Zhong et al., 2021; Zheng et al., 2022).

Clinical trials are still being conducted to assess the effectiveness and safety of these inhibitors in people with breast cancer. In addition, technological developments like the creation of high-throughput screening techniques and computational modelling are making it easier to find and refine new kinase inhibitors for breast cancer treatment. Despite promising results, inhibitors of kinases do have some restrictions. Cancer cells can evolve strategies to resist kinase inhibition, including activation of alternate signalling pathways or mutations in the kinase target, which makes resistance to therapy a prevalent problem (Sledge et al., 2017). Furthermore, the use of kinase inhibitors may be accompanied by toxicities and off-target effects that result in negative side effects and treatment termination (Iancu et al., 2022). As a result, more investigations are required to determine how best to employ kinase inhibitors in the treatment of breast cancer and to find biomarkers that can be used to predict therapeutic response and direct treatment choices. As a result, cutting-edge ways to combat drug resistance are being investigated, including immunotherapies in combination with therapy and precision medicine methods that use tumour genomic analysis to find patient-specific therapeutic targets. In this review, we summarize recent advances in the development of small-molecule inhibitors of kinases for breast cancer therapy, discuss the opportunities and challenges associated with this approach, and highlight areas for future research.

## 2 Kinases as therapeutic targets in breast cancer

Breast cancer involves the activation of various kinases that contribute to the disease’s development and progression. Kinases are key targets for the development of innovative therapeutics because they play a significant role in the progression and initiation of breast cancer. Breast cancer cells may grow, survive, and migrate more easily if their kinase signalling pathways—including serine/threonine kinases, non-receptor tyrosine kinases, and receptor tyrosine kinases—are dysregulated. It has been demonstrated that breast cancer can be effectively treated by focusing on these kinases and the signalling pathways that they activate.

**FIGURE 1:**
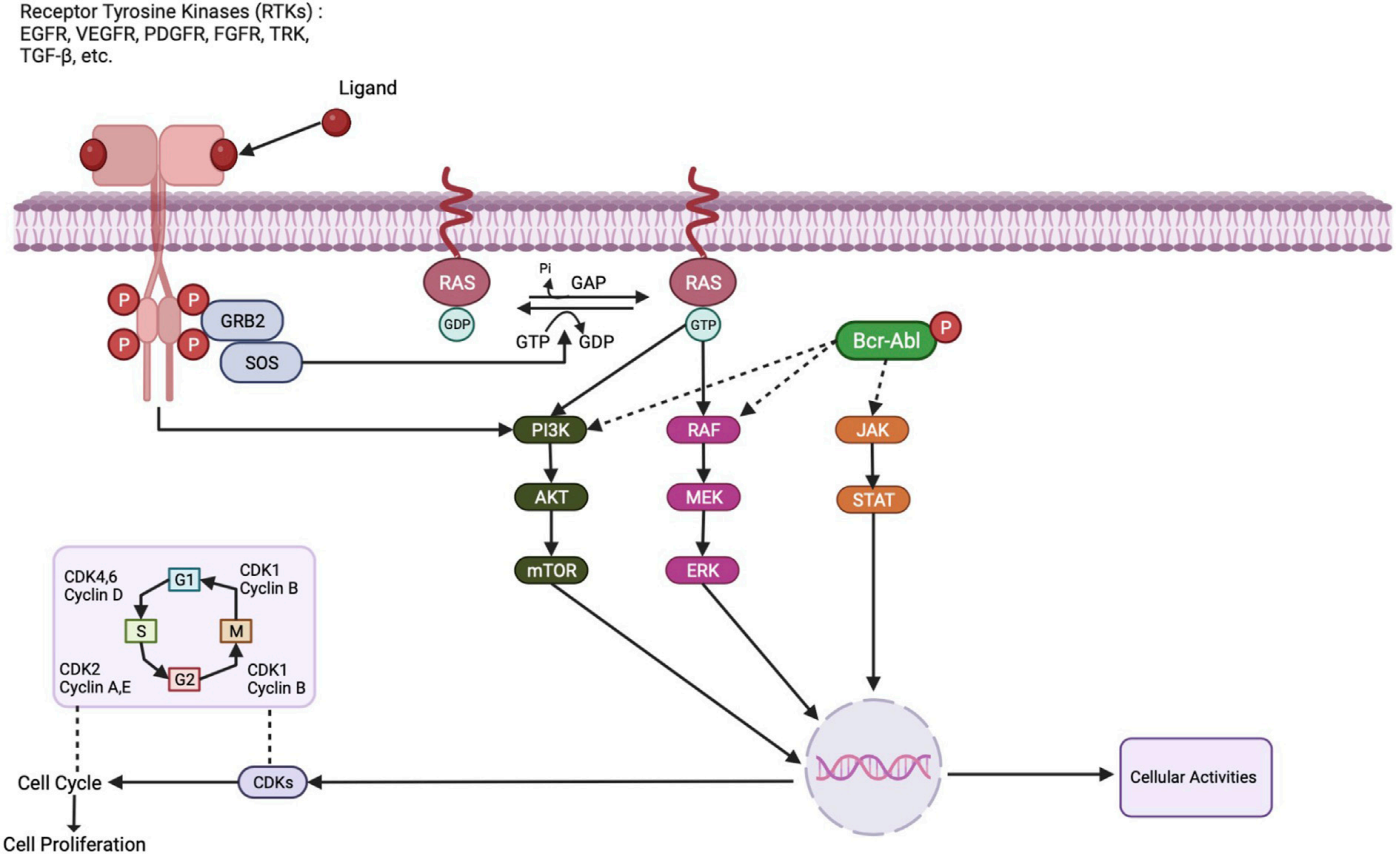
Cellular effects of activation of different protein kinase-dependent pathways.

Genetic and epigenetic changes that activate signalling pathways involved in cell migration, growth, and survival are the primary causes of breast cancer. The activation of downstream signalling pathways that control cell activity is one of them, and receptor tyrosine kinases (RTKs) play a key role by converting signals from external ligands to the intracellular domain as described in Figure 1. Tyrosine Kinase Inhibitors (TKIs) are a class of inhibitors that specifically target tyrosine kinases that are involved in signalling cascades that support cell growth and survival. Lapatinib, a dual TKI that targets the HER2 and EGFR receptors, has proven effective in treating breast cancer that is HER2-positive. In HER2-positive metastatic breast cancer patients, a clinical trial by Blackwell et al. (2012) found a significant increase in progression-free survival (PFS) with lapatinib plus capecitabine compared to capecitabine alone (Blackwell et al., 2010). For instance, members of the EGFR family of receptors, like HER2, are overexpressed in a sizable portion of breast cancer patients and activate several signalling pathways that promote tumour development and metastasis (Hynes and Lane, 2005).

Cell cycle progression is regulated by cyclin-dependent kinases, which are inhibited by Cyclin-Dependent Kinase Inhibitors (CDKIs). The CDK4/6 inhibitor palbociclib has demonstrated promise in the treatment of HR+ HER2-negative breast cancer. In the PALOMA-2 trial, patients with postmenopausal HR+ HER2-negative advanced breast cancer had a better median PFS when palbociclib and letrozole were combined than when letrozole was used alone (Finn et al., 2016). Cyclin-dependent kinase 4/6 (CDK4/6) is one kinase that is frequently overactivated in breast cancer, leading to cell cycle progression by phosphorylating retinoblastoma (Rb) protein. CDK4/6 inhibitors, like ribociclib, palbociclib, and abemaciclib, have shown potential in treating breast cancer by inhibiting cell proliferation and inducing cell cycle arrest (Spring et al., 2019).

The phosphoinositide 3-kinase pathway, a crucial regulator of cell survival and proliferation, is the target of phosphoinositide 3-kinase inhibitors (PI3KIs). Alpelisib, a PI3KI that targets the PI3K alpha isoform, has demonstrated effectiveness in treating advanced breast cancer with PIK3CA mutations. Alpelisib plus fulvestrant extended PFS compared to fulvestrant alone in postmenopausal HR+ HER2-negative advanced breast cancer patients with PIK3CA mutations in the SOLAR-1 trial (André et al., 2019). PI3Ks, which is essential for controlling cell proliferation and survival, is a fundamental regulator of protein kinase B (Akt), which is a downstream effector. In breast cancer, Akt is frequently activated, increasing cell invasion, survival, and proliferation. As a result, targeting protein kinases like Akt is a promising therapeutic approach for the treatment of breast cancer (Manning and Cantley, 2007).

Another kinase involved in breast cancer is the mechanistic target of rapamycin (mTOR), which regulates proliferation, survival, and cell growth. mTOR is commonly activated in breast cancer and is a promising therapeutic target for the disease (Tian et al., 2019). In addition to these, several kinases have been linked to breast cancer, including Janus kinase (JAK), tyrosine kinase-like orphan receptor (ROR), and spleen tyrosine kinase (SYK). For instance, by activating gene expression programs that control cell proliferation, apoptosis, angiogenesis, and metastasis, the JAK/STAT pathway plays a crucial role in breast cancer (Qureshy et al., 2020). The Wnt/-catenin pathway is activated by ROR1 and ROR2, which are overexpressed in numerous breast cancer subtypes, and this promotes tumour growth and metastasis (Menck et al., 2021). SYK, a non-receptor tyrosine kinase, controls breast cancer cell growth and survival and is crucial for B-cell receptor activation (Stewart and Pietenpol, 2001).

Breast cancer typically has overexpressed and activated epidermal growth factor receptors (EGFR), which are receptor tyrosine kinases. As a result of EGFR activation, the mitogen-activated protein kinase (MAPK) pathway and the phosphatidylinositol-3-kinase (PI3K)/AKT/mechanistic target of rapamycin (mTOR) pathway, which promotes cell survival and growth, are both activated as can also be seen in the Figure 1 (Wee and Wang, 2017). Clinical trials for the treatment of breast cancer have shown promise for EGFR inhibitors such as erlotinib and gefitinib (Baselga et al., 2005). Another receptor tyrosine kinase with an overexpression rate of 20% in breast tumours is HER2. When HER2 is activated, downstream signalling pathways that support cell growth and survival including the STAT pathway and the PI3K/AKT/mTOR pathway are also activated (Moasser, 2007). Trastuzumab and lapatinib are two examples of HER2-targeted therapies that have completely changed how HER2-positive breast cancer is treated. Serine/threonine kinases known as CDK4/6 are essential for controlling the cell cycle (Finn et al., 2016). For breast cancer, CDK4/6 inhibitors such as ribociclib and palbociclib have been developed as targeted treatments. These inhibitors are currently authorized for the treatment of hormone receptor-positive, and HER2-negative metastatic breast cancer since they exhibited effectiveness in clinical trials (Braal et al., 2021).

A serine/threonine kinase called BRAF is frequently mutated in malignancies such as melanoma. Studies have revealed that a proportion of breast tumours also have BRAF mutations (Curtis et al., 2012). The MAPK pathway is activated by BRAF mutations, promoting cell growth and survival. Vemurafenib and dabrafenib, two BRAF inhibitors, have demonstrated potential as targeted treatments for BRAF-mutant breast cancer (Davies et al., 2002). Breast cancer frequently harbours mutations in the kinase PIK3CA (Razavi et al., 2018). The PI3K/AKT/mTOR pathway is activated by PIK3CA mutations, promoting cell growth and survival. As targeted treatments for breast cancer, PI3K inhibitors such as alpelisib and taselisib have been created (André et al., 2019). In breast cancer, the non-receptor tyrosine kinase JAK2 is typically overexpressed and activated (Croker et al., 2009). The JAK/STAT pathway, which is involved in cell proliferation and differentiation, can be activated by JAK2. JAK2 inhibitors have been created as specific treatments for breast cancer, including ruxolitinib and fedratinib (Bowman et al., 2000). In breast cancer, the serine/threonine kinase AURKA is typically overexpressed. In addition to being involved in the control of the mitotic spindle assembly checkpoint, AURKA is essential for cell division. Alisertib and barasertib have demonstrated promise as AURKA inhibitors in the treatment of breast cancer (Borah and Reddy, 2021). A critical part of the DNA damage response pathway is played by the serine/threonine kinase CHK1. Breast cancer patients usually have elevated levels of CHK1, which controls DNA repair and cell cycle progression. Prexasertib and LY2606368 are examples of CHK1 inhibitors that have been developed as specific treatments for breast cancer (Kummar et al., 2015). Table 1 summarizes same examples of small-molecule kinase inhibitors commonly used in breast cancer therapy.

**TABLE 1:** Some small-molecule kinase inhibitors used in breast cancer therapy.

S. No	Kinase	Name of inhibitor	Target	Protein substrate	Administration pathway	Type of breast cancer	References
1	Tyrosine kinase	Tucatinib	EGFR and HER	Tyrosine	Oral	Advanced or metastatic HER2-positive	Murthy et al. (2020)
							Sirhan et al. (2022)
2	PI3K	Alpelisib (in combination with fulvestrant)	PI3Kα	Phosphatidylinositol 3-kinase	Oral	Advanced-PIK3CA mutation and HR-Positive	André et al. (2019)
							Narayan et al. (2021)
3	Tyrosine kinase	Neratinib	EGFR and HER	Tyrosine	Oral	HER2-positive	Martin et al. (2017)
							Saura et al. (2020)
4	CDK 4/6	Abemaciclib	CDK4, CDK6	Serine-threonine	Oral	HR-positive, HER2-negative	Goetz et al. (2017)
							Johnston et al. (2020)
							Braal et al. (2021)
5	VEGFR Multi-kinase	Sunitinib	VEGFR, PDGFR, and other tyrosine kinases	Tyrosine	Oral	Metastatic	André et al. (2014)
							Hainsworth et al. (2016)
6	CDK 4/6	Ribociclib	CDK4, CDK6	Serine-threonine	Oral	HR-positive, HER2-negative	Hortobagyi et al. (2016)
							Braal et al. (2021)
7	CDK 4/6	Palbociclib	CDK4, CDK6	Serine-threonine	Oral	HR-positive, HER2-negative	Finn et al. (2015)
							Braal et al. (2021)
8	Tyrosine kinase	Lapatinib	EGFR, HER2	Tyrosine	Oral	Metastatic HER2-positive	Elwaie et al. (2020)
							Xu et al. (2021)

## 3 Small-molecule kinase inhibitors

### 3.1 Mechanisms of action of small-molecule kinase inhibitors

Targeting the ATP-binding pocket of kinases, a highly conserved area in the kinase domain, is how small-molecule kinase inhibitors work. These inhibitors can impede the transfer of phosphate groups from ATP to their substrate proteins by binding to this pocket, which inhibits the activity of kinases and subsequent signalling pathways. Competitive inhibition, non-competitive inhibition, and allosteric inhibition are the three major categories into which small-molecule kinase inhibitors’ methods of action can be divided. For binding to the ATP-binding pocket of the kinase, competitive inhibitors engage in competition with ATP. This kind of inhibition can be reversed, and it is often removed by raising the level of ATP. For instance, the BCR-ABL kinase inhibitor imatinib competes with ATP for binding to the kinase’s ATP-binding site, preventing the activation of the kinase in chronic myeloid leukaemia (CML) cells (Druker et al., 2001). Like ATP, by competing with ATP for binding to the kinase’s ATP-binding site, the small-molecule inhibitor gefitinib of the epidermal growth factor receptor (EGFR) kinase reduces the activity of non-small cell lung cancer (NSCLC) cells (Lynch et al., 2004).

Non-competitive inhibitors decrease the activity of the kinase by causing conformational changes in the kinase protein by attaching to a spot on the kinase that is different from the ATP-binding pocket. This kind of inhibition is permanent and is unaffected by variations in the ATP level. For instance, the Raf kinase inhibitor Sorafenib binds to the kinase’s allosteric site and prevents the activation of the kinase in hepatocellular carcinoma (HCC) cells (Wilhelm et al., 2004). Allosteric inhibitors cause conformational changes in the kinase protein that stop it from functioning by attaching to a location on the kinase that is different from the ATP-binding pocket. Changes in the ATP levels do not affect this sort of reversible inhibition. Lapatinib, for instance, interacts to an allosteric location on EGFR and HER2 kinases and inhibits the activity of these kinases in breast cancer cells (Rusnak et al., 2001).

### 3.2 Developmental strategy for small molecule inhibitors use in therapies

Utilising small molecules that block the tyrosine kinase activity of HER2 receptors is part of the development strategy for targeting HER2 using ErbB pan inhibitors in small molecule inhibitors for breast cancer therapy. These inhibitors prevent tyrosine phosphorylation and subsequent signalling events by competing with ATP at the cytoplasmic catalytic kinase domain. Due to the structural similarities among kinases, maintaining selectivity is essential. HER2 is a preferred partner for the formation of heterodimers in the kinase domain of the ERB family receptors, which exhibit homology. Comparatively speaking, the HER2/EGFR heterodimer is more potent than EGFR homodimers. HER2 kinase is autoinhibited intrinsically and can be activated through allosteric mechanisms via dimer formation, in contrast to other receptor tyrosine kinases (Aertgeerts et al., 2011).

Lapatinib and neratinib are dual-target inhibitors that stop the activity of both EGFR and HER2 and are clinically available for patients with breast cancer. Lapatinib, a reversible TKI, is one of them and is typically used in conjunction with capecitabine to treat advanced or metastatic breast cancers that exhibit HER2 overexpression and have previously been treated with anthracycline, paclitaxel, or Herceptin (Paul et al., 2008). Neratinib is an irreversible inhibitor that is typically prescribed to breast cancer patients who have finished standard Herceptin-assisted therapy and are at high risk of progression but are currently untreated (Deeks, 2017). Additionally, tucatinib (irbinitinib) has an IC50 of 8 nM and is a powerful and selective HER2 inhibitor. This recently approved HER2 inhibitor is also used to treat patients with metastatic or advanced HER2-positive breast cancer who are unable to undergo surgery (Murthy et al., 2020).

### 3.3 Mechanisms of resistance to small-molecule kinase inhibitors

Although small-molecule kinase inhibitors have demonstrated encouraging outcomes in the treatment of breast cancer, medication resistance is a major issue. The target kinase can mutate, alternative signalling pathways can be activated, and the tumour microenvironment can change, among other factors that might lead to resistance to small-molecule kinase inhibitors (Gross et al., 2015). The occurrence of target kinase mutations is one cause of resistance to small-molecule kinase inhibitors. For instance, HER2-targeted medicines like lapatinib and neratinib resistance in breast cancer patients has been linked to alterations in the kinase domain of the HER2 receptor (Rexer and Arteaga, 2012). Likewise, mutations in the oestrogen receptors kinase domain have been linked to resistance to endocrine treatments like tamoxifen and aromatase inhibitors (Michmerhuizen et al., 2020). Resistance to small-molecule kinase inhibitors can also be attributed to the activation of alternate signalling pathways. For instance, resistance to HER2-targeted therapy in breast cancer has been linked to activation of the PI3K/AKT/mTOR pathway (Berns et al., 2007). Moreover, it has been discovered that resistance to HER2-targeted medicines and endocrine therapy is correlated with overexpression of alternative receptor tyrosine kinases, such as MET and IGF-1R (Liu et al., 2009; Osborne and Schiff, 2011).

Breast cancer patients may develop resistance to HER2-directed therapies via a variety of mechanisms. These include downstream signalling, HER2 activating mutations, and mutations that prevent antibody binding—the L755S mutation being a notable example—as well as HER2 reactivation. Upregulation of HER family receptors can result from incomplete HER2 blockade, which can be prevented by combining treatments like pertuzumab and trastuzumab or by using multi-kinase inhibitors that target AXL activation. The cyclin pathway, as well as altered downstream signalling pathways involving genes like PIK3CA and PTEN, can encourage tumour growth (Goutsouliak et al., 2020). Resistance can develop as a result of HER2 and oestrogen receptor crosstalk, which can be avoided by concurrent blockade. Better trastuzumab response has been associated with tumour immune infiltration, particularly higher levels of tumor-infiltrating lymphocytes (TIL) (Kim et al., 2019).

Resistance to small-molecule kinase inhibitors can potentially be influenced by modifications in the tumour microenvironment. For instance, by upregulating alternate pro-angiogenic signalling pathways, the hypoxic tumour microenvironment can facilitate resistance to anti-angiogenic therapy (Carmeliet and Jain, 2011). As a result of several factors, including modifications to the target kinase, activation of alternative signalling pathways, and modifications to the tumour microenvironment, breast cancer can develop resistance to small-molecule kinase inhibitors. The development of new medicines to overcome resistance and enhance outcomes for patients with breast cancer depends on an understanding of these mechanisms of resistance.

With genomic information guiding treatment decisions, selective kinase inhibitors have demonstrated promise in the treatment of cancer. With astounding survival rates, imatinib, for instance, revolutionised the treatment of chronic myeloid leukaemia (CML). Relapse following an initial response, however, continues to be difficult. Several resistance mechanisms have been revealed by genomic analysis of pre- and post-treatment tumours: (a) “on-target” resistance caused by kinase mutations, amplifications, or splicing changes; (b) “on-pathway” resistance involving downstream activation or disruption of negative feedback; (c) bypass mechanisms elevating parallel signalling; (d) epigenetic changes affecting cellular phenotype; and (e) modifications affecting drug transport and stability. These discoveries provide beneficial methods for navigating through opposition (Gross et al., 2015).

### 3.4 Advantages and limitations of small-molecule kinase inhibitors

Small-molecule kinase inhibitors have several benefits and drawbacks that should be carefully examined when choosing a therapy strategy for cancer or other disorders. While they offer tailored therapy and are less invasive than other therapies, their effectiveness can be limited, they can have unintended side effects, and they can be expensive. To create small-molecule kinase inhibitors that are more practical and cheaper for therapeutic usage, more studies are required. Small-molecule kinase inhibitors offer the following advantages:

(i) Targeted therapy: Targeting specific kinases, which are frequently overactive in cancer cells or other disease states, small-molecule kinase inhibitors are made to combat specific diseases. Compared to conventional chemotherapy, this focused strategy lessens the risk of toxicity and minimizes off-target effects (Hoelder et al., 2012).
(ii) Oral administration: Small-molecule kinase inhibitors are often given orally, which is more convenient for patients than intravenous treatment (Roskoski, 2022).
(iii) Less invasive: Compared to conventional cancer therapies like surgery or radiation therapy, small-molecule kinase inhibitors are less invasive. Because of this, they become a more appealing alternative for patients who might not be candidates for more severe treatments (Arora and Scholar, 2005).
(iv) Lower resistance: Compared to other cancer therapies, small-molecule kinase inhibitors have a lower risk of developing resistance. This is because they specifically target molecular pathways that are crucial for the survival and growth of tumours, making it more difficult for cancer cells to build resistance (Fischer, 2017).

Small-molecule kinase inhibitors suffer from following limitations:

(i) Limited efficacy: The efficacy of small-molecule kinase inhibitors can be affected by the existence of mutations or other genetic changes that influence the targeted kinase pathway, and they may not be effective in all patients (Wagle et al., 2014).
(ii) Off-target effects: Small-molecule kinase inhibitors have the potential to cause harmful side effects due to off-target effects. For instance, proteinuria and hypertension can result from certain kinase inhibitors’ blockage of the VEGF receptor (Zhu et al., 2007).
(iii) Development of resistance: Small-molecule kinase inhibitors are less likely than other cancer therapies to cause drug resistance, but cancer cells can nevertheless do so over time by turning on alternate signalling pathways or gaining mutations that render them resistant to the treatment (Wu et al., 2016).
(iv) High cost: Small-molecule kinase inhibitors might be difficult for patients to obtain if they lack enough insurance or financial resources to pay for them because they are usually expensive (Gyawali and Sullivan, 2017).

## 4 Effectiveness and safety analysis of small-molecule kinase inhibitors

For the treatment of breast cancer, small-molecule kinase inhibitors have been the subject of several clinical trials in recent years to assess their effectiveness and safety. In the PALLAS phase 3 trial, patients with HER2-negative, hormone receptor-positive early breast cancer were given the PI3K inhibitor palbociclib in addition to their normal adjuvant endocrine therapy. The fact, that the experiment was abandoned early owing to ineffectiveness, shows that adding palbociclib did not significantly improve the chances of invasive disease-free survival (Mayer et al., 2022). In the DESTINY-Breast01 phase 2 trial, patients with metastatic HER2-positive breast cancer who had previously received pertuzumab, T-DM1, and trastuzumab were assessed for their response to trastuzumab deruxtecan, an antibody-drug combination that targets HER2. In December 2019, the FDA approved trastuzumab deruxtecan after the trial showed notable improvements in overall response rate and progression-free survival (Modi et al., 2020). In the HER2CLIMB phase 2 trial, patients with metastatic HER2-positive breast cancer who had previously received pertuzumab, T-DM1, and trastuzumab were assessed for their response to tucatinib, a selective HER2 inhibitor, in combination with capecitabine and trastuzumab. The FDA approved tucatinib in April 2020 after the trial showed significant improvements in overall survival rate and progression-free survival (Murthy et al., 2020).

In the IMpassion130 phase 3 trial, patients with PD-L1-positive, locally progressed, or metastatic triple-negative breast cancer received either nab-paclitaxel or atezolizumab. When the effectiveness of both treatments was assessed, it was observed that in patients with PD-L1 expression on immune cells, the trial showed significant improvements in progression-free survival and overall survival rate. The FDA then approved nab-paclitaxel and atezolizumab for the treatment of PD-L1-metastatic, positive, triple-negative breast cancer in March 2019 (Schmid et al., 2018). In the MONALEESA-3 phase 3 study, postmenopausal women with advanced HER2-negative, hormone receptor-positive breast cancer, who had not previously received systemic therapy for their condition, were compared to the effectiveness of the CDK4/6 inhibitor ribociclib in conjunction with fulvestrant. The FDA approved the use of ribociclib in combination with fulvestrant in July 2018 because of the trial’s notable improvements in progression-free survival and overall survival (Slamon et al., 2018). In the FALCON phase 3 trial, patients with HER2-negative, hormone receptor-positive advanced breast cancer who had not previously received endocrine therapy for their advanced disease were assessed to determine the efficacy of fulvestrant, an oestrogen receptor antagonist, either in combination or alone with the CDK4/6 inhibitor palbociclib. FDA approved the use of palbociclib and fulvestrant together in combination therapy in February 2016 because the trial showed significant increase in progression-free survival (Finn et al., 2016).

In a randomized phase 2 trial, postmenopausal women with advanced breast cancer who tested positive for the oestrogen receptor and HER2-negative were observed to have significantly increased survival to a combination of palbociclib and letrozole compared to those who received letrozole alone. Moreover, the combination demonstrated moderate toxicity, with neutropenia being the most frequent side effect (Finn et al., 2015). In the MONARCH-3 trial, abemaciclib plus non-steroidal aromatase inhibitors significantly extended progression-free survival in women who had not previously had systemic treatment for advanced disease and who had hormone receptor-positive, HER2-negative breast cancer when compared to placebo plus non-steroidal aromatase inhibitors. The most common adverse response was diarrhoea, and the combination also demonstrated controllable toxicity (Sledge Jr et al., 2020). In the EGF100151 trial, patients with metastatic HER2-positive breast cancer who have previously undergone treatment with anthracyclines, taxanes, and trastuzumab benefited considerably more from lapatinib combined with capecitabine than from capecitabine alone in terms of progression-free survival. Moreover, the combination demonstrated tolerable toxicity; the most frequent side reactions were diarrhoea and hand-foot syndrome (Cameron et al., 2010). In the MONALEESA-2 research, postmenopausal women with advanced hormone receptor positive and HER2-negative breast cancer experienced a significantly longer progression-free survival when using ribociclib plus letrozole. Moreover, the combination demonstrated moderate toxicity, with neutropenia being the most frequent side effect (Hortobagyi et al., 2016). For individuals with early-stage breast cancer who had HER2 positive tumours, neratinib significantly outperformed placebo in the ExteNET study in terms of invasive disease-free survival. However, the incidence of severe diarrhoea and liver damage were greater with neratinib than with placebo (Chan et al., 2016).

## 5 Opportunities and challenges in the development of small-molecule kinase inhibitors

### 5.1 Identification and validation of novel kinase targets

There are possibilities and challenges in the creation of small molecule kinase inhibitors for the treatment of breast cancer. The effectiveness and success of clinical trials can be increased by discovering and validating novel kinase targets using genomics and other methods, as well as by using biomarkers to find the right patient populations. Covalent inhibitors and combination therapy are two techniques for increasing specificity and lowering toxicity that can raise the therapeutic index of kinase inhibitors and lessen negative effects on healthy cells and tissues. The development of small-molecule kinase inhibitors for the treatment of breast cancer requires the identification and validation of novel kinase targets. Using genomic research, such as the Cancer Genome Atlas (TCGA) project, which has uncovered several prospective targets for the treatment of breast cancer, novel kinase targets were discovered (Gentile et al., 2017). The identification of inhibitors with high specificity for new targets can also be accomplished by screening huge libraries of compounds against panels of kinases (Yang et al., 2019). The validation of novel kinase targets can be difficult since it necessitates not only establishing the effectiveness of kinase inhibitors against the target but also comprehending the target’s function in the pathogenesis of breast cancer and identifying biomarkers of inhibitor response (Pushpakom et al., 2019). Moreover, small-molecule kinase inhibitors’ off-target effects can make it challenging to interpret results and identify the precise target responsible for the observed effects (Bhullar et al., 2018).

Functional genomics is an alternative method for determining and validating new kinase targets. This method systematically perturbs genes or proteins to determine which ones are required for cancer cell survival or proliferation (Yang et al., 2019). The mitogen-activated protein kinase 1 (MAP3K1) is one example of a kinase that has been discovered by RNA interference (RNAi) screening to be crucial for the survival of breast cancer cells (Jansen et al., 2017). Finding selectivity for the target kinase while minimizing off-target effects on other kinases or cellular processes is a problem in the creation of small molecule kinase inhibitors, in addition to identifying novel kinase targets. The creation of covalent inhibitors, which irreversibly attach to the target kinase and create a covalent bond that improves selectivity and potency, is one strategy for dealing with this problem (Yang et al., 2019). The use of structure-based drug design is an alternative strategy that entails determining the three-dimensional structure of the target kinase and using computational modelling to create inhibitors that are particular to the target (Bhullar et al., 2018). The selection of the ideal patient group for clinical trials presents another difficulty in the development of small molecule kinase inhibitors. Patients who are more likely to benefit from a particular kinase inhibitor can be found using biomarkers, which are quantifiable signs of a disease’s progression or a treatment’s effectiveness (Ghatalia et al., 2016). For instance, the existence of mutations in the target kinase or the signalling pathways downstream may predict susceptibility to a particular kinase inhibitor (Yang et al., 2019). Moreover, the use of biomarkers in clinical trials can assist in identifying patients who are unlikely to respond to a certain inhibitor, which can increase the effectiveness and success of clinical studies. Small-molecule kinase inhibitor development is also complicated by the possibility of toxicity and unfavourable side effects, especially on healthy cells and tissues. The creation of inhibitors that precisely target kinases that are overexpressed or activated in cancer cells but are barely expressed or dormant in normal cells is one method for overcoming this problem (Bhullar et al., 2018). Combination therapy, which combines the use of several inhibitors or inhibitors with other anti-cancer medicines, can also lower the dosage needed for individual inhibitors and increase their therapeutic index (Ghatalia et al., 2016).

### 5.2 Optimization of small-molecule kinase inhibitors

The achievement of selectivity for the intended target kinase while avoiding off-target effects that could result in toxicity or impair efficacy is a particular difficulty in the optimization of small molecule kinase inhibitors. The use of co-crystal structures of inhibitors bound to target kinases to discover important binding interactions and the use of structure-based design to change inhibitors for enhanced selectivity have both been used as solutions to this problem (Roskoski, 2020). In order to optimize small-molecule kinase inhibitors, it is necessary to overcome potential resistance mechanisms that may develop during therapy. As was previously mentioned, kinase inhibitor resistance can develop through a variety of processes, such as changes in drug metabolism or efflux, activation of alternative signalling pathways, and mutations in the target kinase (D'Angelo et al., 2020). The creation of second-generation inhibitors with increased potency or selectivity, the use of combination therapies that target multiple pathways or mechanisms, and the creation of predictive biomarkers to pinpoint patients who are most likely to respond to therapy are all methods for combating resistance (D'Angelo et al., 2020; Roskoski, 2020). Moreover, the development of efficient drug delivery systems that can increase drug solubility, bioavailability, and tissue distribution is a key component of the optimization of small-molecule kinase inhibitors. This is crucial for medications whose low water solubility, quick metabolization, or quick elimination can restrict their efficacy and bioavailability (Zhou et al., 2020). Creating kinase inhibitors for targets that have proven challenging to treat because of their structural or functional characteristics is another difficulty. Examples of kinases that can be difficult to target with small-molecule inhibitors include those without a clearly defined active site or those that serve as allosteric regulators. Targeted protein breakdown techniques, covalent inhibitors, and fragment-based drug development are some attempts to overcome this problem (Bhullar et al., 2018).

The costly and lengthy process of drug discovery and development is a significant obstacle in the development of small-molecule kinase inhibitors. Significant resources and commitment may be needed for the discovery and confirmation of novel kinase targets, drug candidate optimization, and evaluation of safety and effectiveness in preclinical and clinical research (Roskoski, 2019). Using high-throughput screening techniques to find novel kinase targets, working with academic and business partners to pool knowledge and resources, and creating regulatory frameworks to speed the approval of new medications for patients in need are some approaches to overcoming this obstacle (Roskoski, 2019). Finally, to address unmet clinical requirements and enhance patient outcomes, there is a need for ongoing innovation in the design and manufacturing of small-molecule kinase inhibitors. This includes creating inhibitors for targets that are currently incurable or have a poor prognosis, as well as creating stronger, more focused inhibitors that can enhance therapeutic response and lessen toxicity (Zhou et al., 2020). Overall, the creation of small molecule kinase inhibitors poses a complicated range of difficulties and chances that necessitate cross-disciplinary cooperation and continuous innovation to succeed.

### 5.3 Development of combination therapies for breast cancer treatment

Creating combination therapy with small-molecule kinase inhibitors offers a promising chance to boost the effectiveness of treating breast cancer. Combination medicines reduce the risk of treatment resistance and enhance patient outcomes by focusing on various signalling pathways involved in tumour progression and development (Yap et al., 2013). The use of the chemotherapeutic drug capecitabine along with the small-molecule kinase inhibitor lapatinib is an illustration of a successful combination therapy for breast cancer. Patients with HER2-positive breast cancer who have not responded to treatment have been demonstrated to have improved overall survival and progression-free survival when given this combination (Geyer et al., 2006). The use of the small-molecule kinase inhibitor palbociclib in combination with the hormone treatment medication fulvestrant is another illustration of an effective combination therapy for breast cancer. Patients with advanced breast cancer that expresses hormone receptors but lacks HER2 had improved progression-free survival after receiving this combination (Turner et al., 2015). Combination medicines hold great promise, but their development and application also face formidable obstacles. Finding the best medication combinations that can efficiently target several signalling pathways while reducing side effects and toxicity is a challenge (Lopez and Banerji, 2017).

The creation of reliable preclinical models that can precisely forecast the efficacy and toxicity of combination medicines is another difficulty given the intricate relationships between various signalling pathways, the possibility of drug interactions, and the potential for toxicity. Lastly, continuous clinical trials and translational research are required to assess the effectiveness and safety of combination medicines for the treatment of breast cancer and to find biomarkers that can predict treatment response and help with patient selection (Lopez and Banerji, 2017). Using kinase inhibitors in conjunction with immunotherapy is yet another potential strategy for creating combination treatments. Combining small molecule kinase inhibitors with immune checkpoint inhibitors has demonstrated encouraging outcomes in preclinical investigations for the treatment of breast cancer. For instance, compared to each therapy alone, a study in a mouse model of breast cancer indicated that the combination of a MEK inhibitor and an anti-PD-L1 antibody increased antitumour activity (Ahn and Ursini-Siegel, 2021). Another difficulty is figuring out the best timing and dosage for each drug as well as dealing with any side effects and drug interactions (Finn, 2008). Clinical trials of combination medicines can also be difficult and expensive, and they may call for cooperation between numerous institutions and businesses. Overall, there are possibilities and difficulties in the creation of small molecule kinase inhibitors for the treatment of breast cancer. Targeting kinases has been successful, but toxicities and resistance mechanisms continue to be major problems. New kinase targets must be found, small molecule inhibitors must be optimized, and potent combination therapy must be created in order to overcome resistance and enhance outcomes for breast cancer patients.

### 5.4 Challenges in the clinical development of small-molecule kinase inhibitors

Small-molecule kinase inhibitors have showed a great deal of promise in the treatment of cancer, but their development is fraught with difficulties, particularly in the therapeutic environment. Finding biomarkers that can reliably predict a patient’s reaction to an inhibitor is one of the biggest hurdles. Several clinical trials have failed to show a meaningful clinical benefit for patients due to the dearth of trustworthy biomarkers. This demonstrates the necessity of better patient selection methods and biomarker development (Schmid et al., 2020). The formation of drug resistance presents another difficulty in the clinical development of small molecule kinase inhibitors. Many mechanisms, such as target kinase mutations or the activation of alternate signalling pathways, might lead to resistance. Identification of the resistance mechanisms and the development of countermeasures, such as immunotherapies or combination therapy, are therefore crucial (Lovly and Shaw, 2014). To reduce toxicity while preserving efficacy, small molecule kinase inhibitor dose and schedule must be optimized. Preclinical research and early-stage clinical trials can help with this. Therefore, a deeper comprehension of the pharmacokinetics and pharmacodynamics of the inhibitors is necessary for the creation of the best dose regimens (Hoelder et al., 2012). The complexity of cancer biology and the diversity of patient groups also pose special difficulties in the design of clinical trials for small molecule kinase inhibitors. Innovative trial designs, like adaptive and basket trials, have been proposed to overcome these issues and make patient selection and drug development more effective (Spreafico et al., 2021). To overcome drug resistance, enhance dosing regimens, plan creative clinical trials, and develop better patient selection procedures, researchers, doctors, and regulatory organizations must work together to address these difficulties.

## 6 Conclusion

Application of small-molecule kinase inhibitors for the treatment of breast cancer has gained significant success and attention in recent times. Combination therapy, new pathway targeting, personalized medicine, overcoming resistance, enhancing selectivity, and novel drug delivery technologies will probably be the main areas of future research in this subject. It is envisaged that these initiatives would ultimately result in better results for breast cancer patients as a result of continued technological and scientific advancements. Small-molecule kinase inhibitor research for breast cancer is currently focused on several initiatives, such as the discovery of new targets and the creation of more potent and focused inhibitors. In preclinical and clinical investigations, several small-molecule kinase inhibitors have demonstrated effectiveness in combination with other targeted medicines or chemotherapy. Improved outcomes for breast cancer patients may result from further research into combination therapy. More individualized treatment strategies will be possible with the development of companion diagnostic tests that can pinpoint individuals who are most likely to benefit from specific small-molecule kinase inhibitors. Resistance to small-molecule kinase inhibitors is a significant obstacle in the treatment of breast cancer. Further research into the mechanisms of resistance and the creation of countermeasures may result in the creation of more potent treatments. Small-molecule kinase inhibitors may have unintended consequences that reduce their effectiveness and have negative side effects. The therapeutic index of inhibitors could be improved by making them more focused on a subset of kinases while ignoring others. Drug conjugates and nanoparticle-based drug delivery could boost the efficacy and selectivity of small-molecule kinase inhibitors. Notwithstanding these difficulties, kinase-targeted treatments for breast cancer still have a lot of potential. The development of tailored medicines will continue to be fuelled by the identification of novel targets and biomarkers for breast cancer as a result of advancements in the field of genomics and proteomics.
